# Reply: Overdiagnosis of breast cancer in Denmark

**DOI:** 10.1038/sj.bjc.6601739

**Published:** 2004-03-16

**Authors:** A H Olsen, A Jensen, S H Njor, E Villadsen, W Schwartz, I Vejborg, E Lynge

**Affiliations:** 1 Institut for Folkesundhedsvidenskab, Afdeling for Epidemiologi, Københavns Universitet, Blegdamsvej 3, 2200 København N, Denmark

**Sir**,

As observed by Dr Zahl, it is correct that the cumulative risk in breast cancer incidence increased during the observed period. Indeed, breast cancer incidence has increased steadily in Denmark since the 1960s (Sundhedsstyrelsen, 2003). Assuming linearity in the cumulative risk, we performed a simple least-squares estimation of the trend in breast cancer incidence in the period before screening for both Copenhagen and Fyn. Assuming this trend would have continued if screening had not been introduced, the expected cumulative risk for Copenhagen in 1993–95 was 6.6% and the observed cumulative risk was 6.1% (95% CI 5.3–6.8%). For Fyn the expected cumulative risk for 1996–97 was 5.9%, whereas the observed cumulative risk was 6.6% (95% CI 5.9–7.3%). For both Fyn and Copenhagen the expected cumulative risk was within the 95% confidence interval. Therefore, we see no reason to conclude that overdiagnosis is taking place.

If the underlying increase in the incidence before screening was partly due to opportunistic screening, this would not affect the conclusions since the increase in the number of clinical mammography examinations continued after the introduction of screening (Olsen *et al*, 2003).

It is correct that bringing the time of diagnosis forward should result in a lower incidence in the age group 70–74, and we plan to look at this effect for Copenhagen and Fyn. But whereas entry into the screening programmes is well defined, exit is not. In the second invitation round in Copenhagen, women in the age group 70–71 were invited. In Fyn, women over 70 can participate if they ask for it. This will dilute the effect, and therefore we have not yet performed this analysis.

As mentioned in the article (Olsen *et al*, 2003), inclusion of screen-detected DCIS did not change the picture. Note that the proportion of DCIS detected in the Danish screening programmes is small compared to that in many other programmes, due to a deliberately conservative attitude towards supposedly benign microcalcifications (Vejborg *et al*, 2002).

We will follow the further development of breast cancer incidence in Denmark.
